# Agricultural adaptations to mid-late Holocene climate change in western Türkiye

**DOI:** 10.1038/s41598-023-36109-0

**Published:** 2023-06-08

**Authors:** Tom Maltas, Vasıf Şahoğlu, Hayat Erkanal

**Affiliations:** 1 Institute of Classical Archaeology, University of Vienna, Franz Klein-Gasse 1, 1190 Vienna, Austria; 2 Department of Archaeology, Ankara University, Ankara, Türkiye; 3 Ankara University Research Center for Maritime Archaeology (ANKÜSAM), İzmir, Türkiye

**Keywords:** Climate-change adaptation, Agroecology, Anthropology, Archaeology

## Abstract

The period around the mid-late Holocene transition (*c*. 2200 bc) saw major societal developments across the eastern Mediterranean. At the same time, the region experienced a shift to more arid climatic conditions. This included punctuated episodes of rapid climate change such as the ‘4.2 ka event’, which has been implicated in widespread societal ‘collapse’ at the end of the Early Bronze Age. The ways in which societies adapted agricultural production to cope with a drying climate are poorly understood. We begin to rectify this through stable isotope analysis of archaeobotanical remains from the Aegean region of western Türkiye, conducted to reveal changes in agricultural decision making across the mid-late Holocene transition. We find that Bronze Age farmers adapted agricultural production strategies by investing in drought-tolerant cereals cultivated on drier fields with water management strategies redirected towards pulses. Despite this, we find no evidence for pronounced drought stress in cereals grown during the period of the 4.2 ka event. This raises the potential for alternative explanations for societal disruptions visible across the Anatolian Plateau during this time, such as the breakdown of long-distance trade networks.

## Introduction

The period around the mid-late Holocene transition (*c.* 2200 bc) saw major societal developments across the eastern Mediterranean. The region experienced widespread urbanisation across the third millennium bc, both fostering and linked to developments in metallurgical and craft production and the expansion of long-distance exchange networks^1,2^. New and expansive forms of political control emerged with the growth of institutionalised rulers overseeing territorial polities at varying scales^3,4^. Superimposed against these developments, palaeoclimatic proxies from the region suggest that it experienced an oscillatory decline in winter rainfall from around 5000–4000 bc, culminating in widespread aridity in the late Holocene punctuated by sub-centennial episodes of both wetter and drier conditions^5–8^.

The interaction between these societal and climatic developments is a major focus of research, but one that has primarily been oriented towards understanding the roles of climate change within episodes of societal ‘collapse’^8–10^. The ways in which prehistoric societies adapted to ensure continuity has received less attention. In particular, how prehistoric farmers adapted food production systems to feed increasingly urbanised populations within a drying climate over the long-term is a major question remaining to be addressed. This lacuna is likely due to the limited ability of traditional archaeobotanical approaches to reveal changes in land management strategies and agricultural decision making in the past. Recent developments in stable isotope analysis of archaeobotanical remains have begun to rectify this by providing direct evidence for crop growing conditions in the past, with implications for crop husbandry strategies and the broader nature of agricultural production systems^11–14^. In this paper, we utilise this approach to investigate arable land management practices across the mid-late Holocene transition within a single eastern Mediterranean region, western Türkiye. In doing so, we present one of the first in-depth case studies into how prehistoric farmers adapted agricultural decision making to cope with a drying climate. We also assess the implications of our findings for the impacts of the ‘4.2 ka’ rapid climate change event within the region.

## Inferring crop growing conditions and arable land management practices through stable isotope analysis of archaeobotanical remains

We assess crop growing conditions and arable land management practices through stable carbon and nitrogen isotope analysis. For C_3_ crops such as wheat, barley and pulses, δ^13^C (the ratio of the isotopes ^13^C:^12^C relative to a universal standard) values are influenced by the movement of carbon dioxide through stomata, which is in turn influenced by crop water status, with the greatest discrimination against the heavier isotope ^13^C when stomata are open during periods when water is readily available during carbon fixation^15^. The water status of archaeological grains can be interpreted with reference to both climate and agricultural strategies aimed at preventing water stress^12–14,16^. The relationship between water availability and δ^13^C is strongest in regions where water is a major limiting factor to plant growth. δ^13^C is less sensitive in regions with rainfall averaging over *c.* 550 mm/year^14^. Crop δ^15^N (the ratio of the isotopes ^15^N:^14^N) values largely reflect the soil nitrogen composition when the crop was grown. Crop δ^15^N values may be influenced by a range of factors, such as aridity^17–19^ and exposure to saltwater spray^17,20^, but a major potential factor is anthropogenic soil enrichment, with applications of animal manure found to increase the δ^15^N of arable crops by up to 10‰ based on the amount and frequency^11^. Manuring has a much smaller impact on the δ^15^N values of pulses, which fix nitrogen directly from the atmosphere, but particularly intensive manuring has been shown to elevate pulse δ^15^N values^11^.

While both δ^13^C and δ^15^N are correlated with climate, particularly aridity, anthropogenic inputs within cultivated fields can make it difficult to directly link crop growing conditions to climate. Accordingly, we focus on reconstructing arable land management strategies in order to understand how climate impacted agricultural decision-making rather than crop growing conditions per se.

## Climatic and societal change in western Anatolia around the mid-late Holocene transition

The centuries leading up to the mid-late Holocene transition saw major developments across the Anatolian Plateau and the eastern Aegean islands, which formed part of a cultural *koine* with western Anatolia during this period^21^. At the onset of the Early Bronze Age (EBA/EB) (*c*. 3100/3000 bc), a common settlement plan emerged across the region, with communities organised into longhouses within fortified settlements^22,23^. Large non-domestic structures and central storage entities in some settlements (e.g., Poliochni^24^, Heraion^25^ and Karataş^26,27^) indicate the existence of new social structures with more formalised community cooperation, decision making and leadership than earlier periods^28^.

This trend continued into the late EB II to early EB III (*c*. 2600–2200 bc) at some settlements, which underwent rapid changes in both physical and socio-political organisation to become fortified citadels surrounded by lower towns. The most well-known example from western Anatolia is Troy, where the heavily fortified citadel of Troy II contained a complex of five free-standing megaron buildings up to 40 m in length^29^. The ‘social logic’ expressed by the monumental citadel and deposits of gold and precious metal items (although likely post-dating the abandonment of the central megaron complex) indicate the existence of elite individuals wielding economic and socio-political power on a scale not seen before in the region^28,30^. The foundation of fortified sites at key access points to alluvial plains suggest that elites ruled small territorial polities^3,31^. These developments coincided with the intensification of exchange networks connecting Anatolia and Upper Mesopotamia^1,32^. Imported items and high-status metal objects within Anatolian citadels suggest that control of long-distance exchange networks and the production of metals were key dimensions to elite wealth and power^1,33–35^. Around 600 large storage vessels recovered from the citadel of Troy^36^ also reveal an agricultural basis to elite wealth.

The late EB III saw dramatic disruptions to this cultural landscape. A notable increase in the destruction of settlements between *c*. 2300 and 1950 bc attests to a rapid surge in organised violence^31,35^, after which many sites in western Anatolia were abandoned. In others, communities became smaller and lacked the socio-political structures of the citadel into the early Middle Bronze Age (MBA) (e.g., Troy^37^, Liman Tepe^38^ and Seyitömer^39^, although continuity is evident at Heraion^40^ and possibly Poliochni^41^). Further detail is provided by survey data from the Upper Meander Basin, where the number of sites decreased sharply during the period between 2400 and 1950 bc while larger settlements close to watercourses increased in size^31,42^. This suggests that some communities clustered around larger settlements while others may have shifted to more mobile lifeways that left less substantial traces on the landscape^31^. A divergent trajectory is visible in central Anatolia, where most destroyed settlements were immediately re-established and became increasingly urbanised into the late EB III/early MBA. Elites appear to have sought tighter control of staple products through more complex administration and the centralisation of grain on larger scales^31^.

These disruptions are broadly contemporary with the ‘4.2 ka event’, a short-term shift to drier climatic conditions recorded in palaeoenvironmental records across the eastern Mediterranean and Middle East between *c*. 2300/2200–1900 bc^43–47^. Archaeological evidence for major and broadly synchronous episodes of societal change around this time^48–51^ suggest that it may have been a key factor shaping the cultural landscapes of these regions. Despite this, issues with the chronological resolutions of different palaeoenvironmental and archaeological datasets and variation in regional expressions of global climatic trends^5,46,52^ mean that detailed analysis of high-resolution local datasets is required to robustly demonstrate cause and effect between climatic and societal change within a given region. A map showing the locations of the archaeological sites and palaeoclimatic records discussed here is provided in the supplementary material.

Palaeoenvironmental data from marine cores close to the western coast of Türkiye indicate the onset of increasingly arid climatic conditions from *c.* 3000 BC in the northern Aegean and 2700 bc in the southeast, culminating in the end of a mid-Holocene humid phase around 2300 bc^53–55^. Evidence for a rapid onset, short-term period of aridity that can be linked to the broader 4.2 ka event is more ambiguous. In northwest Türkiye, abrupt lithologic and geochemical changes suggest an extreme drop in the level of Lake Iznik, Bursa, consistent with an intense drought sometime between 2400 and 2200 bc^56^. Further inland, an increase in herb pollen at the expense of conifer forests, an abrupt negative shift in δ^18^O values and a reduction in total organic carbon at various dates but centred around 2200 bc within sediment cores from the Kureyşler Valley, Kütahya, also indicate drier conditions^57^. Both datasets are hindered by low temporal resolution, however, and therefore do not provide clear evidence for a single, punctuated event. Furthermore, a high-resolution speleothem record from Sofular Cave on the Black Sea coast shows no significant trends in δ^13^C values that would indicate drier climatic conditions around 2200 bc^10,58^. In the northern Aegean, δ^13^C values from a high-resolution speleothem record in Skala Marion Cave indicate increasing aridity from 2500 to 2200 bc, but this is not reflected in δ^18^O values^10,59^.

Palaeoenvironmental data from southwest Türkiye are similarly complex. A drop in δ^13^C values from speleothem Ko-1 in Kocain Cave, Antalya, between *c*. 2260 and 2180 bc has been interpreted as evidence for the onset of colder winters resulting in less rainfall^60^. A recent reanalysis of Ko-1 questions this interpretation, however, demonstrating that δ^13^C values are more closely associated with regional fluctuations in effective-moisture/vegetation cover than winter temperature^61^. Speleothem δ^13^C values are negatively correlated with vegetation cover, which is enhanced by greater effective-moisture^61,62^. The drop in δ^13^C values in Ko-1 may therefore indicate wetter climatic conditions around 2200 BC.

Archaeobotanical evidence for the impacts of the 4.2 ka event has been sought at Troy. Stable carbon isotope values of barley grains from Troy IV (*c.* 2200/2150–1950 bc) predominantly fall between *c*. 16.5 and 18‰^63^, consistent with grains of modern barley grown under conditions of moderate to low water availability^12,14^. The average value of barley grains from Troy IV is *c.* 0.5‰ lower than those from Troy II (*c.* 2500–2350 bc), indicating slightly drier growing conditions in the later period. Whether or not this is indicative of drier climatic conditions is, however, ambiguous. Isotopic evidence suggests that barley fulfilled flexible roles within the prehistoric Aegean, variously cultivated on drier soils and/or those receiving both more or less anthropogenic soil enrichment than wheat based on site-specific arable landscapes and farming systems^14,64–69^. The slightly drier growing conditions of barley in Troy IV than Troy II may simply reflect its cultivation on different plots in the later period resulting from flexible patterns of land use.

Palaeoenvironmental data from western Anatolia thus align with datasets across the eastern Mediterranean documenting the gradual onset of more arid climatic conditions across the mid-late Holocene transition. Archaeological developments during this period suggest that farmers successfully adapted agricultural production to a drier climate, facilitating small-scale urbanisation and polity formation. Issues with chronological resolution, conflicting evidence from different proxies and regional environmental variability between available datasets spanning 2300–1900 bc mean that the impacts of the 4.2 ka event in western Anatolia remain unclear, despite the widespread disruptions to settlements visible around this time.

## The study sites

**Figure 1:**
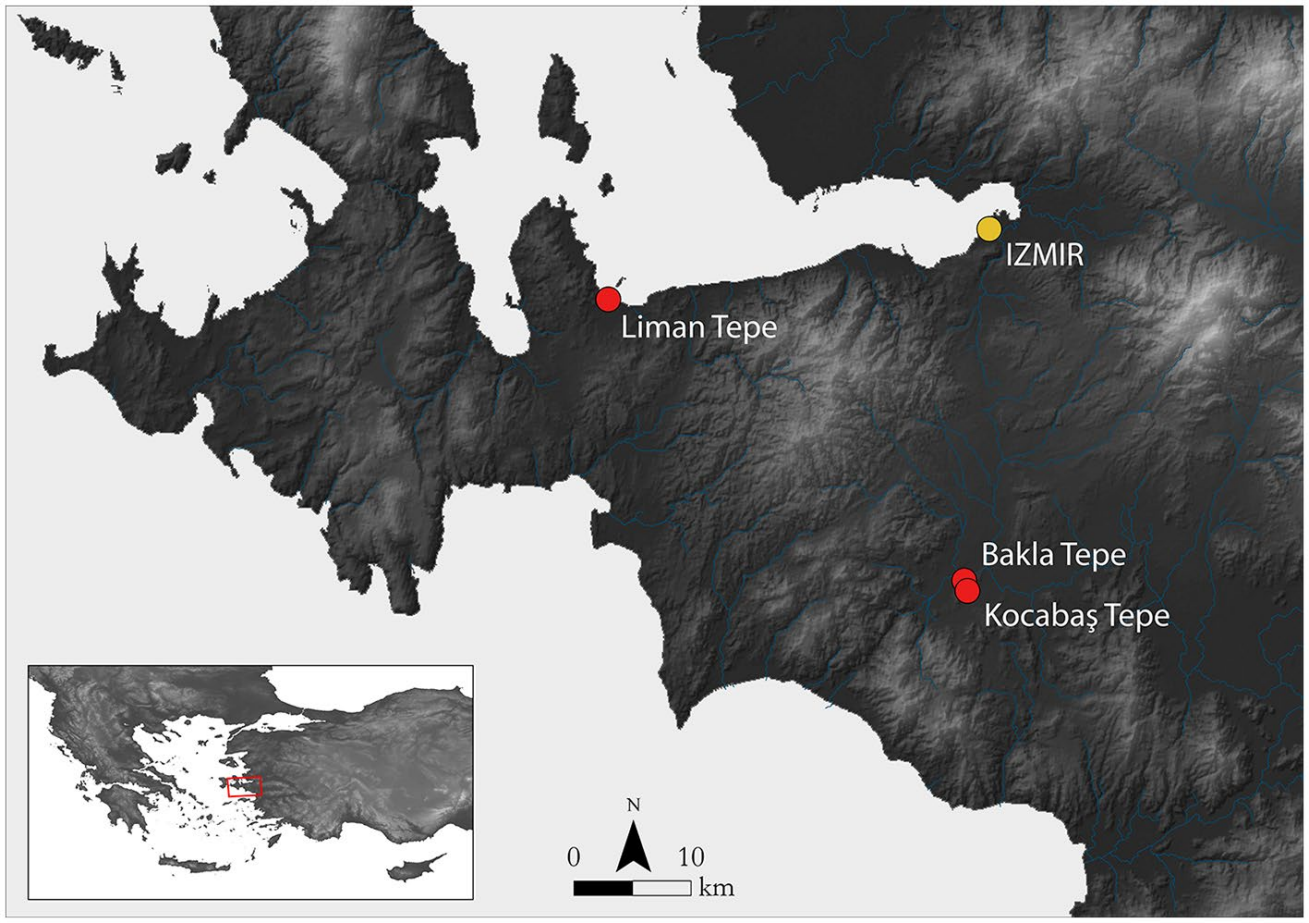
The locations of the sites in our study.

We analysed archaeobotanical remains from the EB I–II to MBA occupations of three sites in the Izmir region of western Türkiye (Fig. 1). These datasets provided a focussed case study into agricultural production within a single region across the mid-late Holocene transition. Below, we outline the key archaeological remains from each of our study sites.

### Liman Tepe

Liman Tepe is a settlement mound located on a peninsula in the Iskele quarter of Urla in the southwest coast of the Gulf of Izmir (Fig. 1). In situ evidence for occupation at the site spans the Middle Chalcolithic until after the end of the Bronze Age^38,70^.

Three phases of occupation are recorded across the EBA, corresponding to the EB I–early EB II (EB I–II; *c.* 3100/3000–2600 bc), late EB II–early EB III (EB II–III; *c.* 2600–2200 bc) and late EB III (*c.* 2200–2000 bc)^38^. The EB I–II settlement is characterised by longhouses sharing walls and abutting a fortification wall. In the EB II–III, the settlement developed into a heavily fortified citadel surrounded by a lower town. Architectural remains within the citadel include an inner ‘fortress’ and a central complex composed of an open courtyard and storage rooms. Finds from the central complex indicate high status and ceremonial activities^38,71^. The use of the central complex ended with a severe fire around 2200 bc as part of the wider pattern of settlement destructions described above. Architectural remains from the succeeding occupation were heavily disrupted by later activity but indicate a small settlement area within the earlier citadel. The early MBA occupation is characterised by oval houses organised along streets and an open courtyard within the earlier citadel^72–74^. A single non-domestic structure was uncovered outside the early EB II bastion and includes a subterranean storage room. Recent radiocarbon dates obtained from charred seeds recovered from the MBA occupation of Liman Tepe date to 2020–1890 cal. bc. The dates are fully reported in supplementary Table 1.

### Bakla Tepe

Bakla Tepe is located in the Cumaovası plain in southern Izmir (Fig. 1). Architectural remains reveal settlements dating to the later part of the Late Chalcolithic (3300–3140 cal. bc), EB I (*c.* 3100/3000–2600/2550 bc) and late EB II–early EB III (EB II–III; *c.* 2600/2550–2200 bc)^75,76^. Like Liman Tepe, the EB I occupation is characterised by a fortified settlement containing longhouses sharing walls and separated into blocks by streets^77^. A cemetery from this period was also identified to the east of the settlement, outside of a fortification wall and ditch. Very few EB II–III architectural remains have been uncovered but a cemetery from this period also was identified to the southeast of the mound^78^. After Bakla Tepe was abandoned, it is thought that occupation moved to the nearby site of Kocabaş Tepe.

### Kocabaş Tepe

Kocabaş Tepe occupies a large rocky outcrop approximately 2 km from Bakla Tepe (Fig. 1). Archaeological remains are known from surveys and limited test excavations^75,79^. Semi-apsidal buildings containing *pithoi* and stone bins associated with carbonised crop remains were uncovered on the south of the mound and have been interpreted as storage rooms. Recent radiocarbon dates obtained from charred seeds recovered from different phases of the storage rooms date to 2140–1970 cal. bc (late EB III/MBA) and 1540–1450 cal. bc (early Late Bronze Age–LBA). The dates are fully reported in supplementary Table 1.

## Results

### Archaeobotanical evidence for crop choice

**Figure 2:**
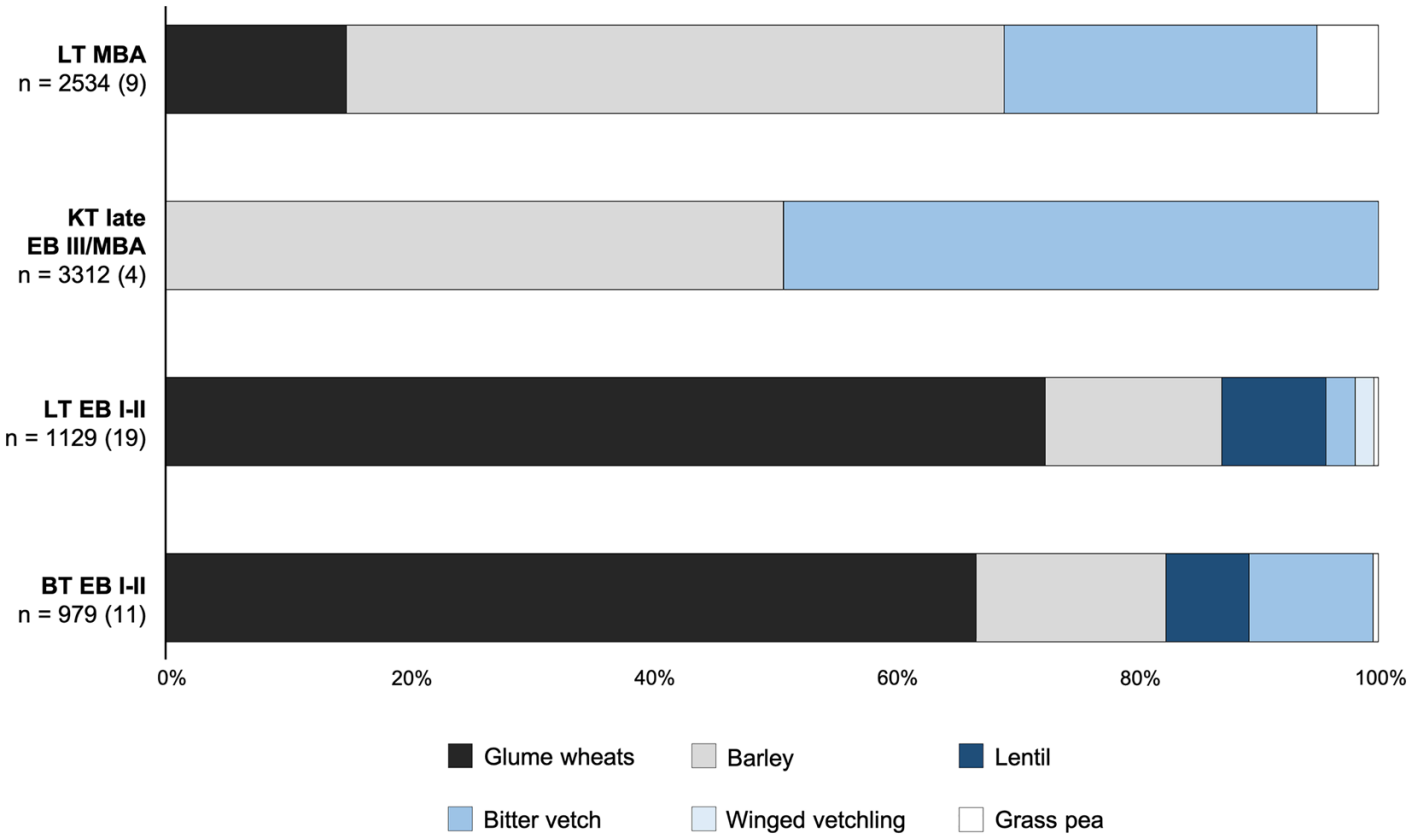
Proportions of major crop taxa recovered from different occupations of the study sites. BT: Bakla Tepe, LT: Liman Tepe, KT: Kocabaş Tepe. n = number of seeds (number of archaeobotanical samples).

Figure 2 compares the proportions of seeds belonging to different crop species in the assemblages from Liman Tepe, Bakla Tepe and Kocabaş Tepe. In the supplementary material, we consider the representativeness of the archaeobotanical samples in our study for the crop spectra from each site/phase. The crop spectra of EB I–II Liman Tepe and Bakla Tepe are very similar, dominated by the glume wheats einkorn (*Triticum monococcum* L.) and emmer (*T. dicoccum* Schrank ex Schübl.) with barley (*Hordeum vulgare* L.) present in lower quantities. Einkorn and emmer were recovered together in all but one sample from both sites and may therefore have been cultivated as a mixed crop and/or processed and stored together. Lentil (*Lens culinaris* Medikus) and bitter vetch (*Vicia ervilia* (L.) Willd.) are the dominant pulses at both sites. Small quantities of winged vetchling (*Lathyrus ochrus* (L.) DC) at Liman Tepe and grass pea (*Lathyrus sativus* L.) at both sites may represent contaminants of other pulse crops. A major shift in the proportions of crop species is visible at both Liman Tepe and in the Cumaovası plain in the late EB III–MBA towards a dominance of barley and bitter vetch. Grass pea also increases in importance at Liman Tepe. While still present in minor quantities within the overall assemblage, grass pea was recovered from a single sample in which it was the most abundant pulse species, suggesting that it may have been cultivated. Free-threshing wheat (*T. aestivum* L.*/durum* Desf.) was recovered in very small quantities within samples dominated by barley from EB III/MBA Kocabaş Tepe, suggesting that it was harvested as a contaminant of barley crops. This is the first time that free-threshing wheat appears in the crop spectra of Kocabaş Tepe or Bakla Tepe, however, raising the possibility of its introduction to the Cumaovası plain as a cultivated crop.

### Isotopic evidence for crop growing conditions and arable land management practices

Summary and full results tables of the stable isotope analysis are provided in supplementary material (Supplementary Tables 3 and 4). The supplementary material also contains an assessment of the reliability of the stable isotope determinations.

#### EB I–early EB II (*c.* 3100/3000–2600 bc)

**Figure 3:**
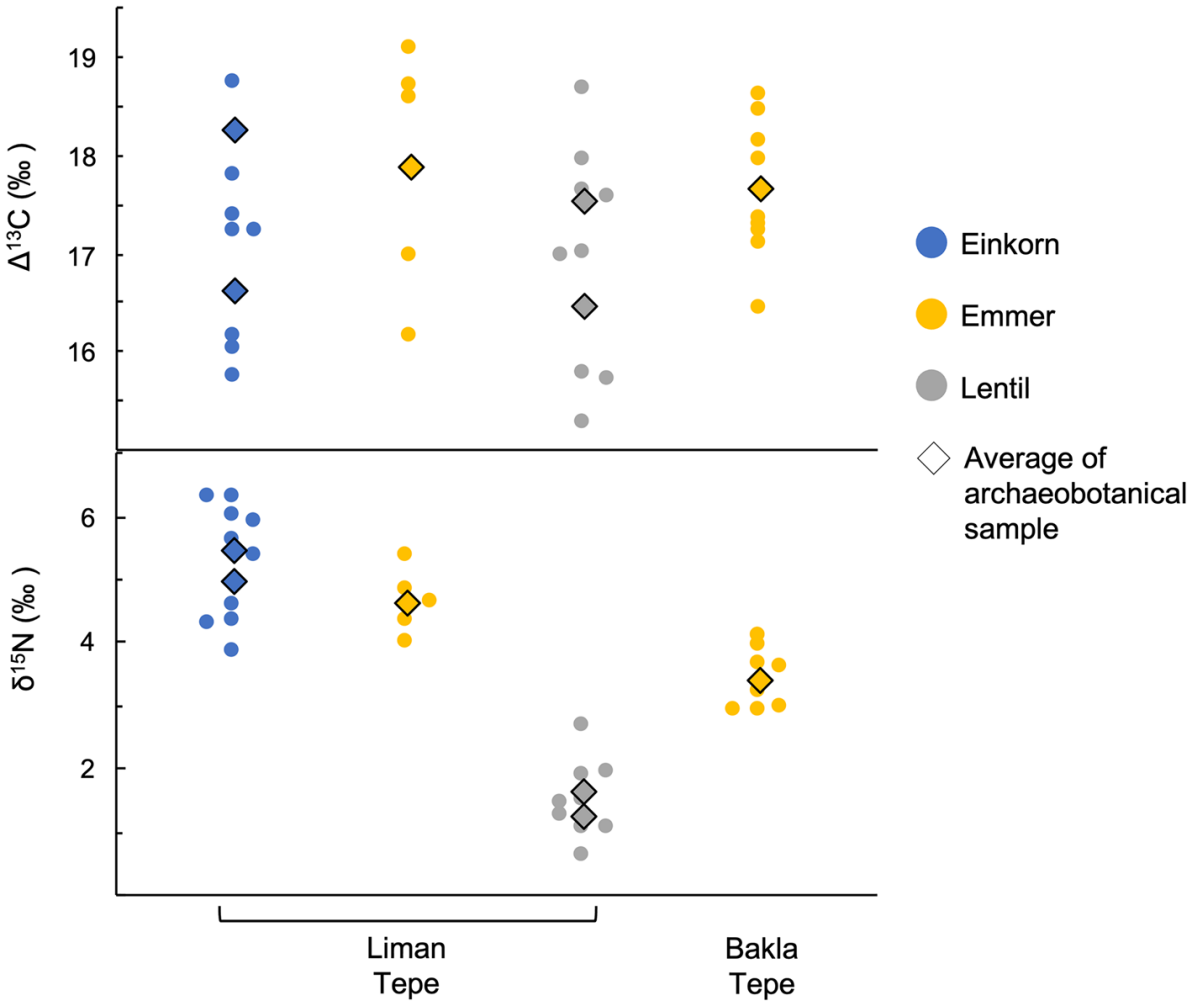
Δ^13^C and δ^15^N values of EB I–II crops from Liman Tepe and Bakla Tepe.

The Δ^13^C values of crops from EB I–II Liman Tepe and Bakla Tepe are shown in Fig. 3. The majority of seeds from both sites have values between 16 and 19‰. While the average annual rainfall of the Izmir region is relatively high (682 mm^80^), meaning that Δ^13^C is less sensitive to water availability, comparison of these values with modern experimental data suggests that it was not a limiting factor to crop growth^12–14^. The Δ^13^C values of einkorn, emmer and lentil from Liman Tepe are not significantly different (Supplementary Table 5), suggesting that they were grown under comparable watering regimes.

The δ^15^N values of crops can be interpreted with reference to potential soil enrichment through applications of organic waste such as manure^11,81^. Other potential causes of elevated soil δ^15^N are aridity^17–19^ and soil salination from sea-spray^17,20^. Palaeoclimatic studies suggest that precipitation in the Izmir region was slightly higher than today in the third millennium bc^6^. Under these conditions, it is unlikely that aridity would be a cause of elevated δ^15^N and unmanured cereals would be expected to have similar values to those grown in temperate Europe^11,81^. Indeed, some Late Chalcolithic (3190–3130 cal. bc) cereal grains from Liman Tepe have δ^15^N values close to 0‰^69^ (Fig. 5), suggesting that they were grown on soils with little to no δ^15^N enrichment^11^. We may therefore infer that the δ^15^N values for non-enriched soils at the sites in our study lie between 0 and 2.1‰, the latter being the range of δ^15^N values of single grains of modern barley grown in the same unmanured field^82^. This is consistent with values below 2.5‰ for modern unmanured crops from central Europe^11^. Sea-spray is a potential cause of enrichment at Liman Tepe due to its proximity to the coast. Cereals exhibit high δ^13^C values in saline rich environments^83^, meaning that δ^15^N enrichment from salt water may be visible in a positive correlation between δ^13^C and δ^15^N values. δ^13^C and δ^15^N values of cereals from Liman Tepe are not significantly correlated (Pearson correlation, r = 0.167, p = 0.303). Crop δ^15^N values from the sites in our study may therefore be interpreted with reference to anthropogenic soil enrichment. δ^15^N values of EB I–II glume wheats from Liman Tepe and Bakla Tepe are elevated above 2.5‰ and are therefore consistent with anthropogenic soil enrichment^11,81^ (Fig. 3). The values of einkorn and emmer wheat from Liman Tepe are not significantly different (Supplementary Table 5). Nitrogen in manure becomes available to crops over the course of several years, meaning that the comparable δ^15^N values between the two species are the result of long-term similarities in soil enrichment^11^. Coupled with the comparable Δ^13^C values, this supports the inference that they were cultivated as a mixed crop, continuing a practice from the Late Chalcolithic occupation of Liman Tepe^69^.

The average δ^15^N values of lentil from Liman Tepe are between 1.5 and 2‰. Pulses assimilate nitrogen from the atmosphere and are less affected by soil enrichment than cereals^11^. Modern pulses that received particularly intensive manuring in Euboea, southern Greece, were found to have values ranging from 1‰ to above 4‰^11^. Those grown without manure had values predominantly below 1‰. Low level enrichment elevates pulse δ^15^N values within the range of measurement error and is indistinguishable from unenriched values^11^. Lentil δ^15^N values from Liman Tepe are therefore consistent with high levels of soil enrichment.

#### Late EB III–MBA (2140–1890 cal. bc)

**Figure 4:**
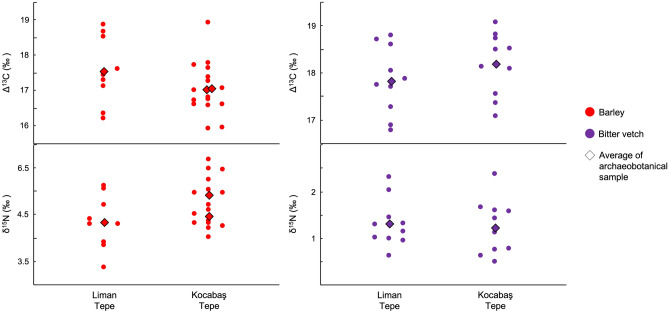
Δ^13^C and δ^15^N values of late EB III–MBA crops from Liman Tepe and Kocabaş Tepe.

The Δ^13^C values of crops from late EB III/MBA Kocabaş Tepe and MBA Liman Tepe are shown in Fig. 4. The values of barley from each site largely fall between 16 and 18‰. Comparison with modern experimental data suggests that some crops may have experienced drought stress while water was not a limiting factor to the growth of others^12–14^. The values of bitter vetch from each site are consistent with well-watered pulses. Δ^13^C values of bitter vetch from both sites are significantly higher than barley, indicative of wetter growing environments, when the latter is offset for a 1‰ difference between modern barley and pulses grown under the same conditions of water availability^12^ (Supplementary Table 5). When offset just − 0.5‰, barley Δ^13^C values are significantly lower than bitter vetch at Liman Tepe but not Kocabaş Tepe. Δ^13^C values of barley from Kocabaş Tepe remain *c.* 0.8‰ lower than bitter vetch when offset − 0.5‰, however.

Direct comparison of the Δ^13^C values of cereals and pulses is complicated by the different growth cycles of the two crop types. Cereals produce all of their ears at one time and, in the Mediterranean, grain filling coincides with the onset of drought^84^. In contrast, pulses produce successive pods throughout the crop cycle meaning that seeds may develop earlier and under higher water status than cereal grains. This may have contributed to the higher Δ^13^C values of bitter vetch than barley from our study sites. Despite this, the higher water status of bitter vetch than barley when compared with the modern experimental data of their respective crop types (i.e., when the Δ^13^C values of bitter vetch and barley are not directly compared) suggests that bitter vetch was grown on more well-watered plots. Furthermore, the δ^15^N values of bitter vetch from both sites have average values above 1.5‰, consistent with intensive soil enrichment of pulses. Without ample water supply, intensive soil enrichment can damage crops^85^, suggesting that bitter vetch would have been grown on sufficiently well-watered plots. Barley δ^15^N values from both sites are elevated above 2.5‰ and are consistent with some degree of non-intensive anthropogenic soil enrichment.

## Discussion

**Figure 5:**
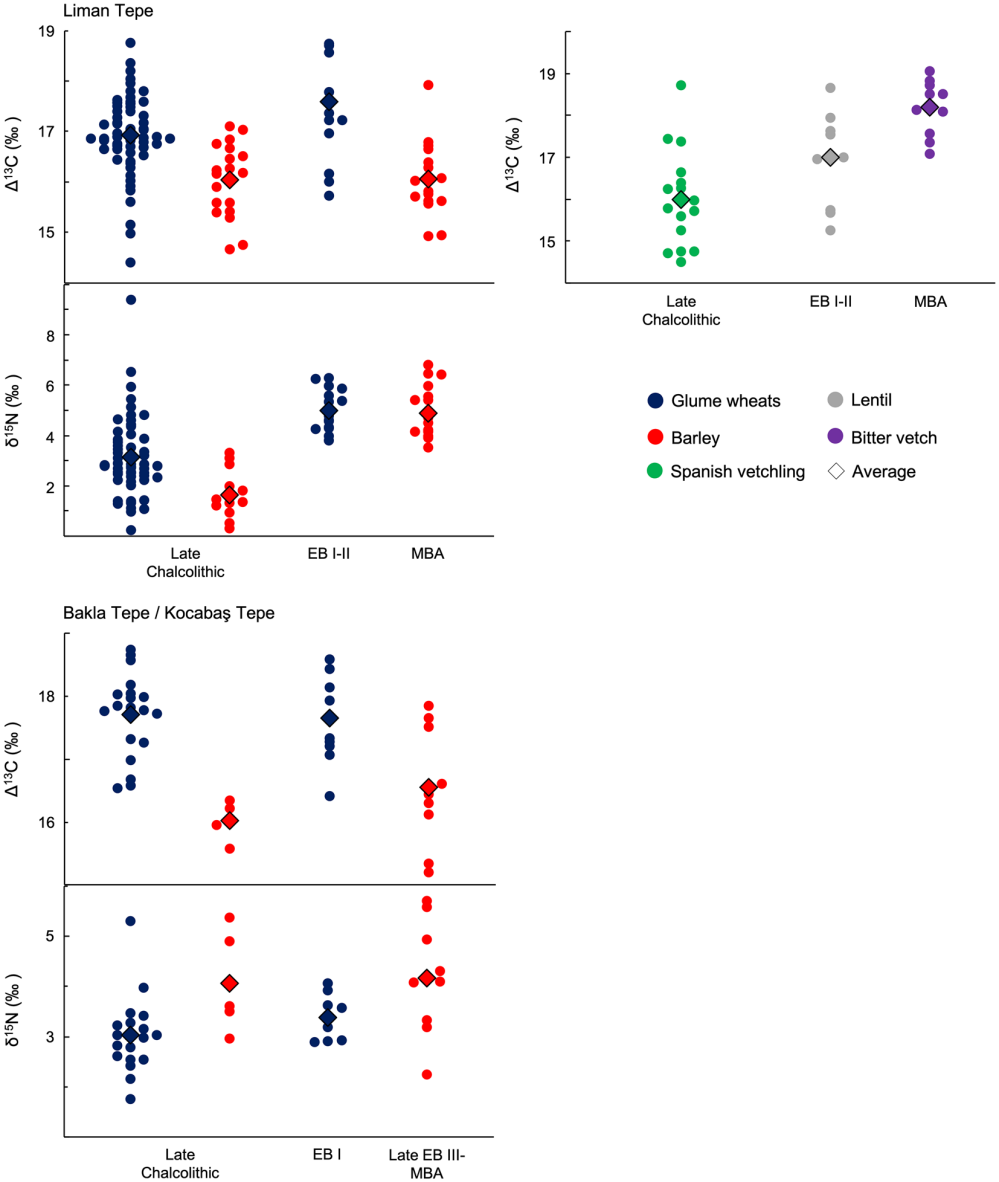
Δ^13^C and δ^15^N values of Late Chalcolithic, EB I–II and late EB III–MBA crops from the sites in our study. Barley Δ^13^C values are offset for a 1‰ difference between wheat and barley grown under the same conditions.

Archaeobotanical and isotopic data have revealed key differences in crop husbandry strategies in the Izmir region spanning the mid-late Holocene transition. To investigate this further, Fig. 5 compares the stable isotope results for EB I–II and late EB III–MBA crops from the sites in our study, alongside those from Late Chalcolithic Liman Tepe and Bakla Tepe published in an earlier study^69^.

The shift towards a dominance of barley in the early MBA crop spectrum of Liman Tepe suggests that it became the primary cereal crop during this period. This is supported by its comparable δ^15^N values to EBA glume wheats (Supplementary Table 5), contrasting with the lower soil enrichment of barley than glume wheats in the Late Chalcolithic (Fig. 5). The Δ^13^C values of MBA barley remain comparable to those of Late Chalcolithic barley and significantly lower than those of EBA glume wheats when offset by both − 1 and − 0.5‰ (Supplementary Table 5), however, suggesting that increased soil enrichment was not matched by an increase in crop water status. In contrast, pulse Δ^13^C values increase significantly between the EB I–II and MBA. While this may result in part from the slightly earlier fruiting time of bitter vetch than lentil in our study region^86^, it also suggests that pulses were grown on more well-watered soils in the MBA than EB I–II. The average Δ^13^C value of EB I–II lentil is also *c.* 1‰ higher than Late Chalcolithic Spanish vetchling, but the values of lentil fall within the range of those of Spanish vetchling and the two groups are not significantly different.

While the isotopic data must be interpreted with caution, the results of our study point to a shift in cultivation strategies between the EB I–II and early MBA at Liman Tepe towards an investment in cereals cultivated under drier conditions and the redirection of well-watered soils and/or water management strategies towards pulses. The lower Δ^13^C values of barley than glume wheats at Late Chalcolithic Liman Tepe reflect an awareness of its ability to withstand drier growing environments, strongly suggesting an investment in drought-tolerant cereal cultivation at the onset of the MBA. Both bitter vetch and grass pea are also known as relatively drought-tolerant pulse crops^87,88^, but it is not clear whether this contributed to agricultural decision-making at Liman Tepe.

In the Cumaovası plain, the dominance of barley at late EB III/MBA Kocabaş Tepe is also matched by comparable δ^15^N values to EB I–II glume wheats from Bakla Tepe, but, unlike Liman Tepe, also Late Chalcolithic barley (Fig. 5). This is due to the more intensive manuring of barley than glume wheats at Late Chalcolithic Bakla Tepe, plausibly as a source of early pasture on which to graze livestock^69^. Like Liman Tepe, however, the Δ^13^C values of EB III/MBA barley from Kocabaş Tepe are significantly lower than those of EB I–II glume wheats from Bakla Tepe when offset − 1‰ (Supplementary Table 5). This difference is not significant when barley is offset just − 0.5‰, but the average value of EB III/MBA barley remains 0.54‰ lower than EB I–II glume wheats. Like Liman Tepe, the cultivation of barley on drier soils than glume wheats at Late Chalcolithic Bakla Tepe reflects a history of exploitation for its drought tolerance within the Cumaovası plain, suggesting an investment in drought tolerance in late EB III/MBA agricultural production.

The evidence thus suggests that farmers in the Izmir region responded to the onset of drier climatic conditions around the mid-late Holocene transition by orienting arable farming systems towards drought tolerance. While the annual precipitation of Izmir is relatively high for the Aegean and Anatolia, it varies significantly year to year, with a standard deviation of 141 mm across the mid-twentieth century^80^. Monthly rainfall is similarly unpredictable, with the probability of an extremely dry month across mid-late twentieth century calculated as 27%^89^. This can be particularly detrimental considering the marked seasonality of rainfall in the region, rendering winter crops (such as those in our study) dependent on heavy rainfall in the winter and early spring. Reductions in winter rainfall indicated by palaeoclimatic proxies in the Aegean may therefore have fostered the adaptations shown here. The broad temporal span of our dataset (up to 700 years) prevents inferences regarding the agency of individuals within this process, but the adaptive flexibility of recent farmers in the Mediterranean is well-attested^85^ and beginning to be recognised in the archaeological record^90^.

The EB III–MBA archaeobotanical remains in our study fall within the period of the 4.2 ka event, raising the potential of a direct local response to the broader climate event. The limited chronological resolution of the archaeobotanical dataset and the issues with regional palaeoenvironmental records outlined above make this difficult to assess. Elsewhere in western Anatolia, archaeobotanical remains from the citadels of Troy and Küllüoba attest to the continuation of EB I–II crop spectra into the EB II–III^91,92^. If extrapolated to the Izmir region, this would suggest that agricultural adaptations took place between the EB II–III and EB III–MBA, placing them more firmly within the period of the 4.2 ka event. Palaeoenvironmental data from western Anatolia highlight the variability of climatic conditions between regions, however, meaning that further archaeobotanical data from the Izmir region itself are necessary to more accurately establish the timing of local agricultural adaptations.

Whether Bronze Age farmers responded to a long or short-term shift towards aridity becomes less significant when we note that we find no evidence for pronounced drought stress in late EB III–MBA barley. Also reflected in the isotopic data from EB III Troy^63^, this aligns with Δ^13^C values of cereal grains in the Middle East that indicate less dry conditions in coastal than inland regions around 2200 BC, following modern patterns of mean annual rainfall^93^. This suggests that the increased aridity across the mid-late Holocene transition/4.2 ka event was mild enough and/or agricultural adaptations successful enough to prevent pronounced drought stress in crops. While long-term reductions in yield resulting from drier growing conditions may have threatened the agricultural basis of elite wealth in citadels such as Troy, this raises the potential for more indirect causes for the social and political instability visible across well-watered parts of western Anatolia at the time of the 4.2 ka event. A plausible explanation lies in the importance of long-distance exchange networks to elite wealth and power in the EB II–III, the breakdown of which is recorded around 2200 bc and is likely to have linked the fortunes of western Anatolian citadels to the more arid regions of Anatolia and the Middle East where the impacts of the 4.2 ka event are well-attested^1,31,33,93^.

## Conclusion

This study has presented some of the first evidence for how farmers in Anatolia and the Aegean adapted crop growing conditions and agricultural decision-making in response to the onset of drier climatic conditions across the mid-late Holocene transition. They did this by investing in drought-tolerant cereals cultivated on drier fields with water management strategies redirected towards pulses. Analysis of further samples from the EB II–III occupation of Liman Tepe is necessary in order to establish the potential role of the 4.2 ka event in agricultural decision-making within the region. It is significant, however, that we find no evidence for pronounced drought stress in cereals dating to the proposed period of the event. This raises the potential for more indirect causes for the social and political instability visible across western Anatolia at the time of the 4.2 ka event, such as the breakdown of long-distance exchange networks connecting Anatolia and the Middle East. Ultimately, while more datasets are needed to elucidate this picture further, our results contribute evidence for the likely diverse ways in which past populations in the eastern Mediterranean adapted to a changing climate.

## Methods

### Archaeobotanical sampling, identification and analysis

Archaeobotanical samples were taken from visible concentrations of charred plant remains and/or burnt deposits. Samples were analysed using a low-power stereo microscope (7–45 ×) at the Ankara University Research Center for Maritime Archaeology (ANKÜSAM), Izmir. Samples were analysed if they contained a minimum of 20 crop seeds. A threshold of 20 identifiable items was chosen as the minimum amount considered to reflect the relative proportions of taxa within a sample to a reasonable degree of accuracy and reliability. The full methods of sub-sampling, identification and analysis of archaeobotanical remains follow those outlined in Maltas et al.^94^.

### Sampling and preparation of crop seeds for stable isotope analysis

The sampling strategy for stable isotope analysis is described in the supplementary material. Seeds were selected for analysis if they showed little to no morphological distortion and had dense interior structures consistent with charring within the ‘optimal charring window’ of 215–260 °C^95,96^. We identified the presence of soil-derived contaminants within the selected seeds using Fourier transform infrared spectroscopy with attenuated total reflectance (FTIR-ATR). FTIR spectra were baseline corrected using Agilent Resolution Pro and compared to grains experimentally contaminated by Vaiglova et al*.*^66^. The results are described in the supplementary material. We chose not to pre-treat seeds prior to stable isotope analysis, but we gently scraped them to remove any surface contamination visible under × 7–45 magnification. Following Kanstrup et al*.*^97^, we aimed to analysis 10 seeds from each archaeobotanical sample. Where this was not possible, we analysed a minimum of three seeds from each sample. This resulted in a total of 87 grains from 13 archaeobotanical samples from the three sites. The number of samples analysed from each site and occupation phase are outlined in supplementary Table 6.

### Stable isotope analysis

Individual seeds were crushed using an agate mortar and pestle and weighed into tin capsules for IRMS analysis on a Sercon EA-GSL mass spectrometer. Raw isotope ratios were calculated using an internal alanine standard. Three-point normalisation of δ^13^C values to the Vienna Peedee Belemnite scale (VPDB) and δ^15^N values to atmospheric N2 was conducted using the standards alanine, seal collagen and cow collagen. EMA P2 and leucine were used as check standards. Measurement uncertainties were calculated as the combined uncertainty of the raw measurement and reference standards, following Kragten^98^. Calculations were carried out using the statistical programming language R (3.2.3). The average measurement uncertainties for δ^13^C were 0.27‰ for Liman Tepe, 0.25‰ for Bakla Tepe and 0.27‰ for Kocabaş Tepe. For δ^15^N, they were 0.49‰ for Liman Tepe, 0.36‰ for Bakla Tepe and 0.47‰ for Kocabaş Tepe.

The δ^13^C and δ^15^N values of archaeobotanical seeds were corrected for the effects of charring by subtracting 0.11‰ and 0.31‰, respectively. These are the average offsets between crop grains that are uncarbonised and those that were experimentally charred at a range of intervals and temperatures that resulted in undistorted seeds with dense interior structures^94^. Archaeological δ^13^C values were converted into Δ^13^C in order to compare them with modern reference stable isotope determinations. Δ^13^C value were calculated from the determined δ^13^C values (δ^13^C_plant_) and a δ^13^C_air_ value approximated by the AIRCO2_LOESS system^84^. The equation follows Farquhar et al*.*^15^:

$${\Delta }^{13}\mathrm{C}=\frac{{\delta }^{13}{\mathrm{C}}_{\mathrm{air} }- {\delta }^{13}{\mathrm{C}}_{\mathrm{plant}}}{1 + {\delta }^{13}{\mathrm{C}}_{\mathrm{plant}}/1000}$$

### Statistical analysis

Statistical analyses were carried out using SPSS 28. Two-tailed oneway ANOVA and post-hoc Tukey’s tests were used to compare more than two groups with equal variance. Two-tailed Kruskall–Wallis and post-hoc Dunn tests were used to compare more than two groups with non-equal variance. Two groups were compared using t-tests. Equality of variance was identified using Levene’s test.

## Supplementary Information

Supplementary Information 1.

Supplementary Table 4.

## Data Availability

All of the isotopic data generated and analysed during this study are included in the main article and/or supplementary information. The archaeobotanical data are presented as a summary figure. The raw archaeobotanical data will be made available in a digital repository.
