# Supplementary material for: Agricultural adaptations to mid-late Holocene climate change in western Türkiye
Source: Sci Rep. 2023 Jun 8;13:9349. doi: 10.1038/s41598-023-36109-0 (PMC10250297; doi:10.1038/s41598-023-36109-0)
Supplement: Supplementary file 1 — Supplementary Information 1. [file 41598_2023_36109_MOESM1_ESM.docx]

Agricultural adaptations to mid-late Holocene climate change in western Türkiye

Supplementary material

Tom Maltas^1^, Vasıf Şahoğlu^2,3^ and Hayat Erkanal†^2,3^

^1^Institute of Classical Archaeology, University of Vienna, Vienna, Austria
^2^Department of Archaeology, Ankara University, Ankara, Türkiye
^3^Ankara University Research Center for Maritime Archaeology (ANKÜSAM), Izmir, Türkiye
*Corresponding author: Institute for Classical Archaeology, University of Vienna, Franz Klein-Gasse 1, 1190 Wien
[tom.maltas@univie.ac.at](mailto:tom.maltas@univie.ac.at)

**
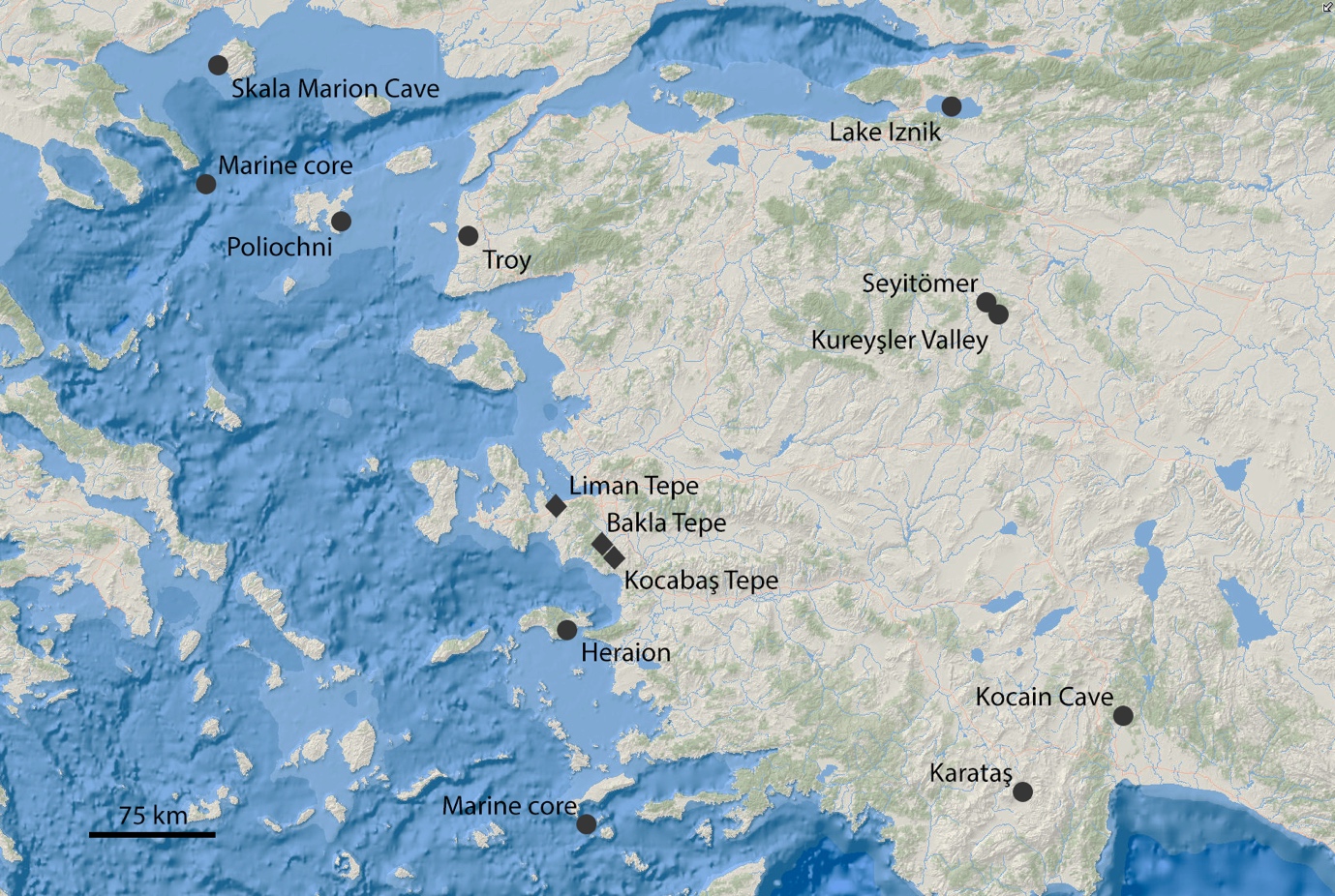
**

**Supplementary Figure 1.** The locations of the sites and palaeoenvironmental records discussed in the text. Basemap adapted from ArcGIS Online.

**Radiocarbon dates**

| **Sample** | **Site** | **Material** | **Context** | **Date BP** | **Cal BC (2σ)** |
| --- | --- | --- | --- | --- | --- |
| OxA-38865 | Liman Tepe | Barley grain | Storage room | 3607 ± 17 | 2026-1898 |
| OxA-38866 | Liman Tepe | Barley grain | MBA fill | 3596 ± 17 | 2022-1892 |
| OxA-38867 | Liman Tepe | Barley grain | MBA fill | 3599 ± 17 | 2022-1894 |
| OxA-38870 | Liman Tepe | Bitter vetch seed | Oval house floor | 3603 ± 19 | 2026-1893 |
| OxA-38860 | Kocabaş Tepe | Bitter vetch seed | Around storage *pithos* | 3669 ± 17 | 2137-1974 |
| OxA-38861 | Kocabaş Tepe | Bitter vetch seed | Refuse pit | 3682 ± 17 | 2139-1981 |
| OxA-38862 | Kocabaş Tepe | Barley grain | Floor of storage room | 3245 ± 17 | 1536-1449 |

**Supplementary Table 1.** Radiocarbon dates from Liman Tepe and Kocabaş Tepe. Dates were calibrated using OxCal 4.4, IntCal 20^1,2^ using atmospheric data from Reimer *et al*.^3^

**Results**

**Archaeobotanical evidence for crop choice.** The number of archaeobotanical samples and range of contexts sampled from the sites in our study are shown in supplementary Table 2. The proportions of seeds of different crop species recovered from the occupations of each site are likely to have been shaped by a range of taphonomic processes. The samples from EB I-II Liman Tepe contain a mix of plant types (e.g., annual crops, fruit and nut taxa), structures (e.g., grain and chaff) and species. This suggests that they represent the debris of crops stored and/or processed for consumption. In many cases, this is supported by their recovery from the interior occupation horizons of longhouses. The samples from EB I-II Bakla Tepe derive from various sources. Those from EB I-II longhouses have mixed compositions and also likely represent domestic waste. Other samples derive from EB I-II graves and may have been used within funerary rites^4^. These are primarily composed of clean cereal grains and smaller amounts of pulse seeds. One sample derives from a later EB II deposit and is composed entirely of pulse seeds. Deposits of domestic waste are contexts into which plant remains enter from a range of activities, meaning that they are more likely to capture the range of plant taxa utilised by households than individual storage deposits^5^. At Bakla Tepe, this is evidenced by the full range of plant species found in the graves and EB II deposit also being recovered from the EB I-II longhouses. The EBA crop spectra within our study therefore provide a good indication of the range of staple crop species utilised at the sites.

Three samples from MBA Liman Tepe derive from an oval house and three derive from the subterranean storage rooms of the non-domestic structure built outside the early EB II bastion. Those from the oval house were taken from two near pure deposits of barley and bitter vetch and likely represent stored crops. Those from the subterranean storage room derive from a large concentration of barley and also likely represent a storage deposit. The remaining three samples from MBA Liman Tepe contain fewer remains of more mixed composition and may represent domestic waste. One of the samples derives from a hearth and is therefore likely to represent domestic waste swept into a fire. The samples from late EB III/MBA Kocabaş Tepe derive from near pure deposits of crop seeds in storage rooms and are therefore also likely to be the remains of stored crops.

Storage deposits provide selective evidence for the range of staple crop species utilised within a settlement as other cultivated species may have been stored elsewhere and therefore not represented in the available samples. Indeed, the majority of the glume wheat grain from MBA Liman Tepe was recovered from the samples that likely represent domestic waste. As noted in the main text, the small quantities of free-threshing wheat at Kocabaş Tepe likely entered the archaeobotanical assemblage as contaminants of barley crops but may also have been cultivated on the Cumaovası plain. Despite this, the absence of a corresponding storage deposit of glume wheats in the Liman Tepe oval house and the large-scale storage of barley within the dedicated storage rooms of both Liman Tepe and Kocabaş Tepe strongly suggest a focus on the cultivation of this species during the late EB III-MBA occupations of these sites. This contrasts sharply with the secondary status of barley in the EB I-II occupations of Liman Tepe and Bakla Tepe. This contrast is maintained when the probable waste deposits from MBA Liman Tepe are considered alone. Barley makes up 46% of cereal grains within these samples, compared with 17% of cereal grains within the EB I-II samples.

| **Site** | **Period** | **No. of samples** | **Contexts sampled (number of samples)** |
| --- | --- | --- | --- |
| Liman Tepe | MBA | 9 | Oval house (3), subterranean storage room (3), hearth (1), misc. fills (2) |
|  | EB I-II | 19 | Longhouse floor (9), burnt soil (1), misc. fills (9) |
| Bakla Tepe | EBA | 11 | Longhouse hearth/floor (2), workshop floor (1), burials (5), misc. EB I fill (2), EB II fill (1) |
| Kocabaş Tepe | Late EB III/MBA | 4 | Pit (2), ashy deposit (1), around *pithos* (1) in storage room |

**Supplementary Table 2.** The number of archaeobotanical samples and range of contexts sampled from the sites in our study.

**Isotopic evidence for crop growing conditions and arable land management practices.** A summary of the results of the stable isotope analysis are presented in supplementary Table 3. The full results are presented in supplementary Table 4. We assessed the reliability of the stable isotope determinations by comparing their C:N ratios to modern cereals and pulses charred by Fraser *et al*.^6^. Similar C:N ratios between the modern and archaeological grains suggest that the original δ^13^C and δ^15^N of the latter were not perceptibly distorted by

|  |  | **Species** | **High** | **Low** | **Average** | **Standard deviation** | **Range** |
| --- | --- | --- | --- | --- | --- | --- | --- |
| **Liman Tepe** | | | | | | | |
| EB I-II | δ^13^C (‰) | Einkorn | -21.87 | -24.76 | -23.19 | 0.95 | 2.89 |
|  |  | Emmer | -22.29 | -25.08 | -24.04 | 1.06 | 2.79 |
|  |  | Lentil | -21.43 | -24.69 | -23.04 | 1.04 | 3.27 |
|  | δ^15^N (‰) | Einkorn | 6.29 | 3.82 | 5.23 | 0.87 | 2.47 |
|  |  | Emmer | 5.33 | 3.97 | 4.56 | 0.50 | 1.36 |
|  |  | Lentil | 2.65 | 0.61 | 1.47 | 0.57 | 2.04 |
| MBA | δ^13^C (‰) | Barley | -22.07 | -24.95 | -23.15 | 0.69 | 2.88 |
|  |  | Bitter vetch | -23.19 | -25.09 | -24.24 | 0.60 | 1.89 |
|  | δ^15^N (‰) | Barley | 6.83 | 3.51 | 5.01 | 1.00 | 3.32 |
|  |  | Bitter vetch | 2.35 | 0.49 | 1.23 | 0.56 | 1.87 |
| **Bakla Tepe** | | | | | | | |
| EB II | δ^13^C (‰) | Emmer | -22.54 | -24.62 | -23.68 | 0.65 | 2.08 |
|  | δ^15^N (‰) |  | 4.05 | 2.88 | 3.46 | 0.47 | 1.17 |
| **Kocabaş Tepe** | | | | | | | |
| Late EB III/MBA | δ^13^C (‰) | Barley | -22.34 | -24.88 | -23.64 | 0.87 | 2.54 |
|  |  | Bitter vetch | -22.22 | -26.04 | -24.25 | 1.03 | 3.81 |
|  | δ^15^N (‰) | Barley | 5.71 | 2.25 | 4.16 | 1.07 | 3.46 |
|  |  | Bitter vetch | 3.06 | 1.27 | 2.19 | 0.58 | 1.79 |

**Supplementary Table 3.** Summary of the results of the stable isotope analysis. Values have been corrected for a charring offset of -0.11‰ for δ^13^C and -0.31‰ for δ^15^N.

post-depositional processes and can be reliably obtained. We compared the ratios of archaeological cereals to modern einkorn, emmer and hulled barley. No data were available for modern bitter vetch, so we compared the archaeological pulse seeds to modern lentil, pea and broad bean. The C:N ratios of the archaeological cereals ranged from 16.7 to 41.3. The modern cereals ranged from 17.9 to 28.2. The archaeological pulses ranged from 9.1 to 14.9. The modern pulses ranged from 8.8 to 13.1. The C:N ratios of archaeological and modern cereals are significantly different (two-tailed t-test, *p* = .006). This is likely due to high C:N ratios in a number of barley grains from Kocabaş Tepe and Liman Tepe (supplementary Figure 2), resulting from low %N (averaging 1.9%). The δ^15^N values of these grains fall within the range of those with C:N ratios comparable to modern grains (supplementary Figure 2), however, suggesting that low %N did not distort grain δ^15^N values.

The C:N ratios of archaeological and modern pulses are also significantly different (two-tailed t-test, *p*, = <.001). This is likely due to differences in the C:N ratios of bitter vetch and the modern reference taxa. The C:N ratios of archaeological lentil are not significantly different to the modern pulses (two-tailed t-test, *p* = .956), but the majority of the archaeological bitter vetch grains have higher ratios than the modern pulses (supplementary Figure 2). The δ^15^N values of bitter vetch are comparable to those of lentil, however (supplementary Figure 3), suggesting that this did not distort the isotopic results.

Following Szpak and Chiou^7^, we also plotted the δ^15^N values against the C:N ratio of archaeological grains in order to assess the presence of patterns consistent with post-burial alteration of δ^15^N values^8^. Shown in supplementary Figure 3, no correlations were apparent. This suggests an absence of post-burial alterations of δ^15^N values.

C:N

%N

%N

C:N

Emmer

Einkorn

Bitter vetch

Barley

Lentil

Modern cereals

Modern pulses

EB I-II

EB III-MBA

**Supplementary Figure 2.** C:N ratios of archaeological seeds plotted against %N compared to modern cereals and pulses charred by Fraser *et al*.^6^.

C:N

δ15N

Liman Tepe


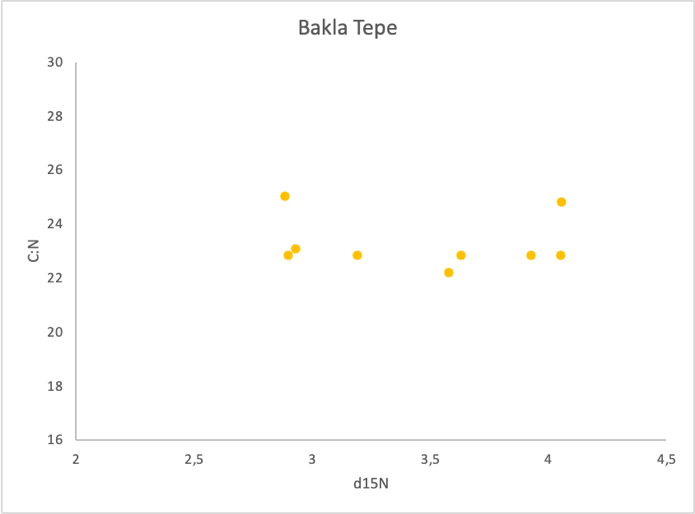


Bakla Tepe

C:N

δ15N

Emmer

Einkorn

Bitter vetch

Barley

Lentil

EB I-II

EB III-MBA

C:N

Kocabaş Tepe

δ15N

**Supplementary Figure 3.** C:N ratios of archaeobotanical seeds plotted against δ^15^N.

| **Material** | | **Δ^13^C** | | | **δ^15^N** | | |
| --- | --- | --- | --- | --- | --- | --- | --- |
|  | | ***F* ^*^** | **t ***** | ***p* value** | ***H* ^**^** | **t ***** | ***p* value** |
| **Liman Tepe** | |  |  |  |  |  |  |
| EB I-II einkorn vs. emmer | |  | (11) = -1.37 | .199 |  | (12.65) = 1.69 | .116 |
| EB I-II glume wheats vs. lentil | | (4, 31) = .621 |  | .891 |  |  |  |
| MBA barley vs. bitter vetch | Barley Δ^13^C -1‰ | (5, 53) = 30.67 |  | **<.001** |  |  |  |
|  | Barley Δ^13^C -0.5‰ | (5, 53) = 20.86 |  | **<.001** |  |  |  |
| MBA barley vs. EB I-II glume wheats | Barley Δ^13^C -1‰ | (8, 74) = 12.04 |  | **.012** | (8) = 62.16 |  | .855 |
|  | Barley Δ^13^C -0.5‰ |  | (27) = 2.29 | **.030** |  |  |  |
| EB I-II lentil vs. Late Chalcolithic Spanish vetchling | | (15, 200) = 7.40 |  | .311 |  |  |  |
| MBA bitter vetch vs. EB I-II lentil | |  | (17) = -2.95 | **.009** |  |  |  |
| **Bakla Tepe / Kocabaş Tepe** | |  |  |  |  |  |  |
| KT barley vs. bitter vetch | Barley Δ^13^C -1‰ | (5, 53) = 30.67 |  | **.008** |  |  |  |
|  | Barley Δ^13^C -0.5‰ | (5, 53) = 20.86 |  | .246 |  |  |  |
| KT late EB III/MBA barley vs.  BT EB II emmer | Barley Δ^13^C -1‰ |  | (17) = 2.80 | **.012** | (8) = 62.16 |  | .169 |
|  | Barley Δ^13^C -0.5‰ |  | (16) = 1.35 | .195 |  |  |  |

*ANOVA and post hoc Tukey’s performed. **Kruskal-Wallis and post hoc Dunn’s performed. ***t-test performed.

**Supplementary Table 5.** Results of the statistical tests conducted on the isotope results. Significant results are shown in bold. KT = Kocabaş Tepe, BT = Bakla Tepe.

**Methods**

**Sampling and preparation of crop seeds for stable isotope analysis.** We sampled grains for stable isotope analysis from a range of archaeological contexts (supplementary Table 6). From EB I-II Liman Tepe, we sampled grains of einkorn and emmer and seeds of lentil from the floors of two longhouses. Samples from MBA Liman Tepe were taken from concentrations of barley and bitter vetch within an oval house and from a large deposit of barley within the subterranean storage structures built outside the early EB II bastion. No differences in δ^13^C or δ^15^N values were present between barley grains from the oval house and subterranean storage structures, so we did not present the contexts separately in our study. From Bakla Tepe, we sampled emmer grains from an EB II pit containing cut into the EB I settlement. Barley grains from late EB III/MBA Kocabaş Tepe were sampled from around a storage *pithos* and from a pit within a storage room.

We identified the presence of soil-derived contaminants within archaeological seeds using Fourier transform infrared spectroscopy with attenuated total reflectance (FTIR-ATR). Seeds were subsampled for FTIR-ATR from each site and occupation period from which we took samples for stable isotope analysis. In supplementary Figure 4, the FTIR spectra of archaeobotanical grains/seeds from the sites in our study are compared to modern grains experimentally contaminated by Vaiglova *et al*.^9^. Surface contaminants on archaeobotanical grains were not removed prior to the FTIR analysis. A minor source of humic contamination is indicated by peaks at 1080 and 1010 cm^-1^ in the spectra. Large peaks at 1080, 1010 and 3690 cm^-1^ in the spectra of sediment scraped off grains from Kocabaş Tepe and Bakla Tepe indicate the presence of humics, suggesting that sediment on the surface of the grains was the source of humic contamination. Grains were therefore not pre-treated but were gently scraped to remove any surface contaminants visible at x7-45 magnification.

| **Site** | **Period** | **Context** | **No. archaeobotanical samples** | **No. grains/ seeds** |
| --- | --- | --- | --- | --- |
| Liman Tepe | MBA | Oval house | 2 | 16 |
|  |  | Subterranean storage room | 1 | 13 |
|  | EB I-II | Floor longhouse 2 | 3 | 15 |
|  |  | Floor longhouse 3 | 3 | 14 |
| Bakla Tepe | EB II | Refuse pit | 1 | 9 |
| Kocabaş Tepe | Late EB III/ MBA | Around *pithos* in storage room | 1 | 10 |
|  |  | Refuse pit in storage room | 1 | 10 |

**Supplementary Table 6.** The number of grains that underwent stable isotope analysis from the different contexts and periods of occupation of the sites in our study.


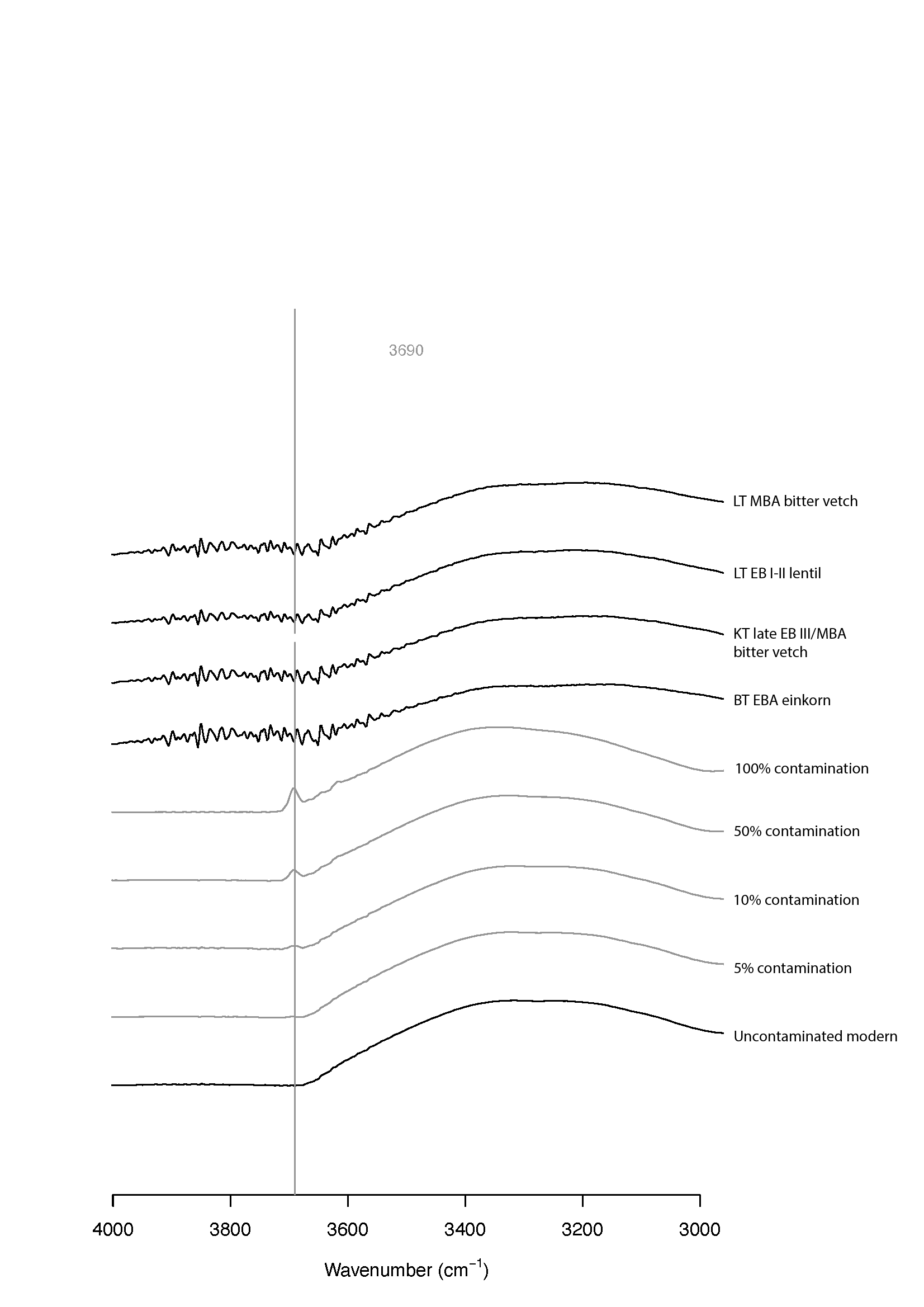

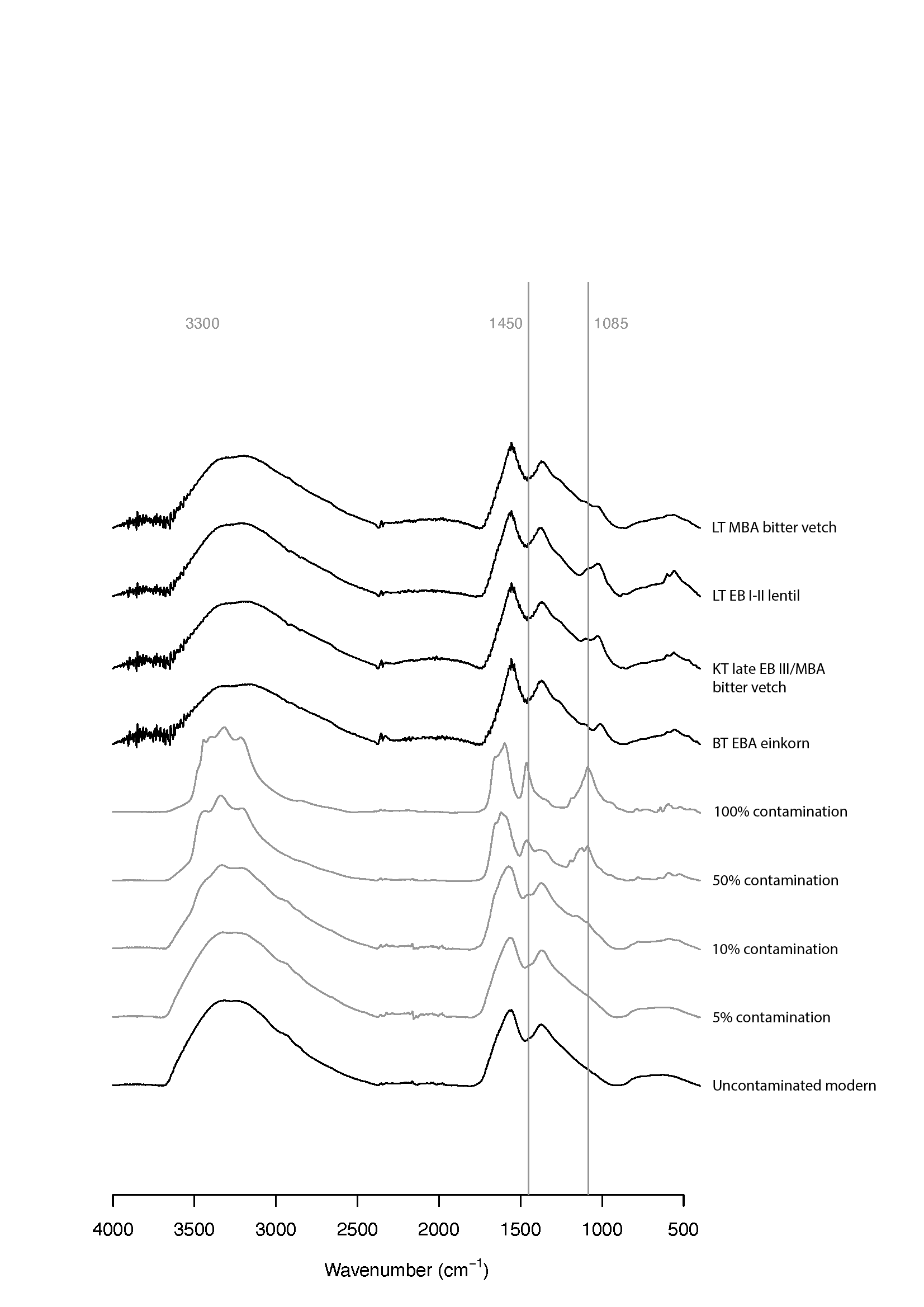

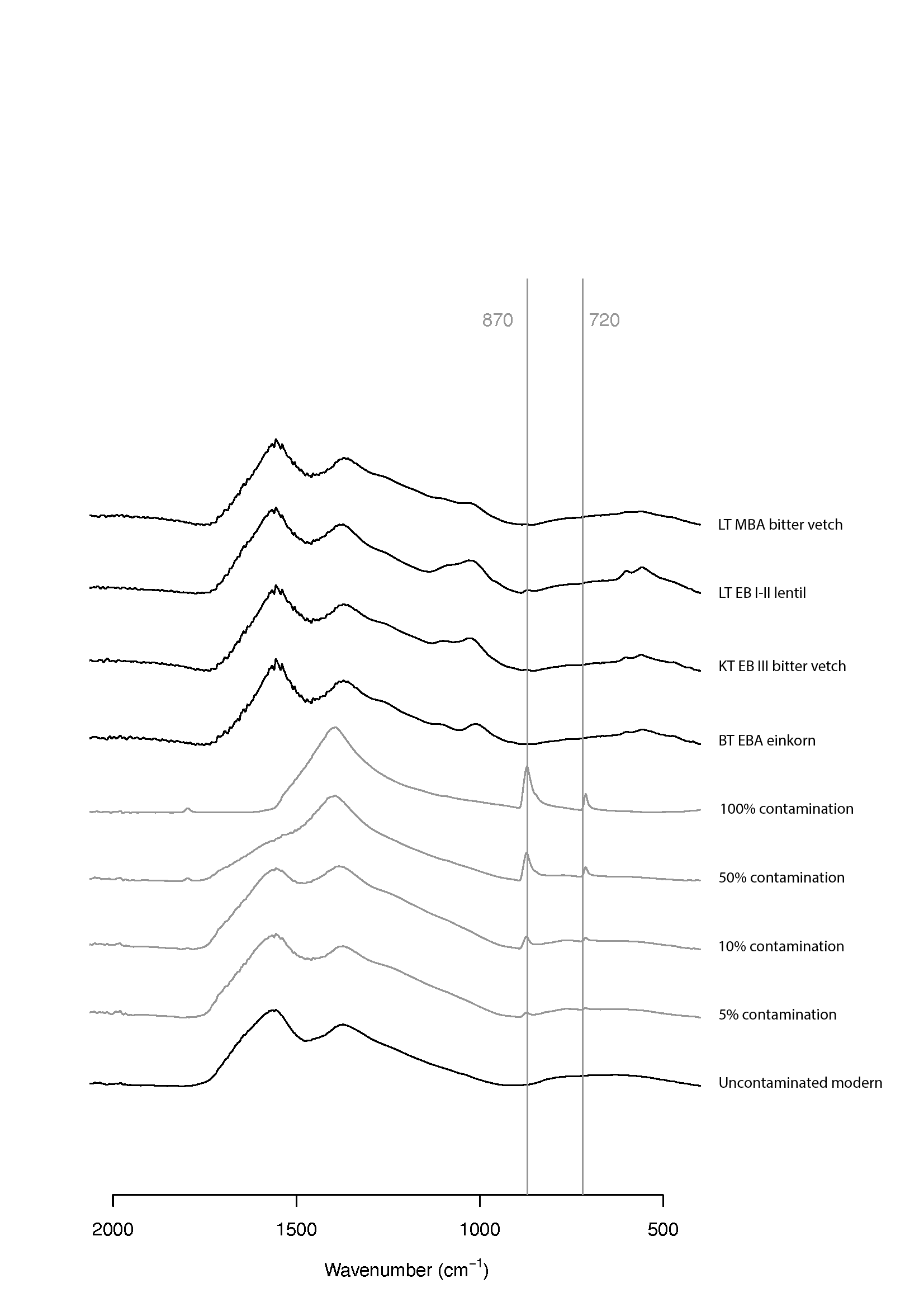


Carbonates

Nitrates


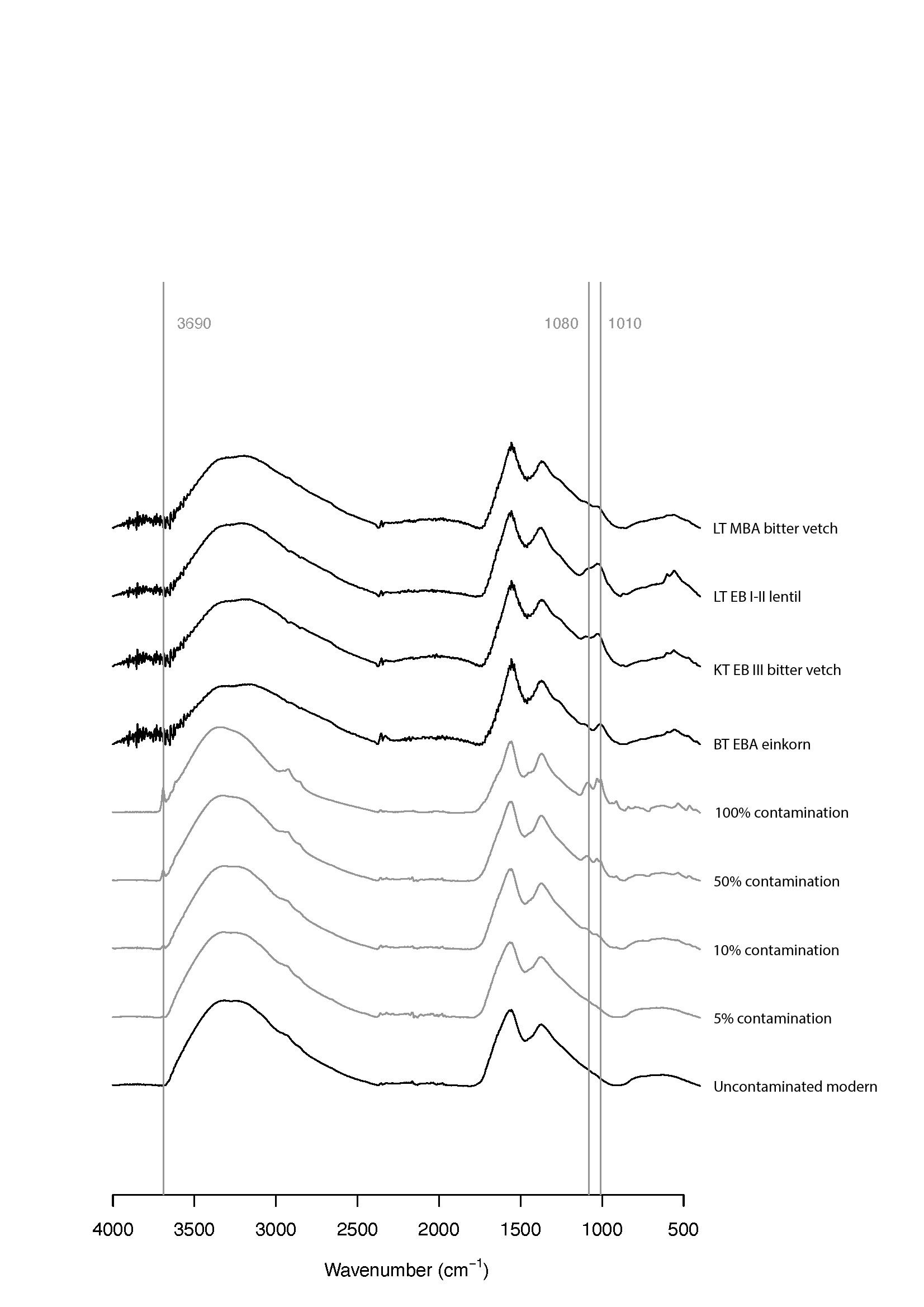


Humics

Humics

**Supplementary Figure 4.** The FTIR spectra of archaeobotanical crop seeds and sediment from the sites in our study compared to modern grains experimentally contaminated by Vaiglova *et al*.^9^. LT – Liman Tepe, BT – Bakla Tepe, KT – Kocabaş Tepe. Figure continued below.


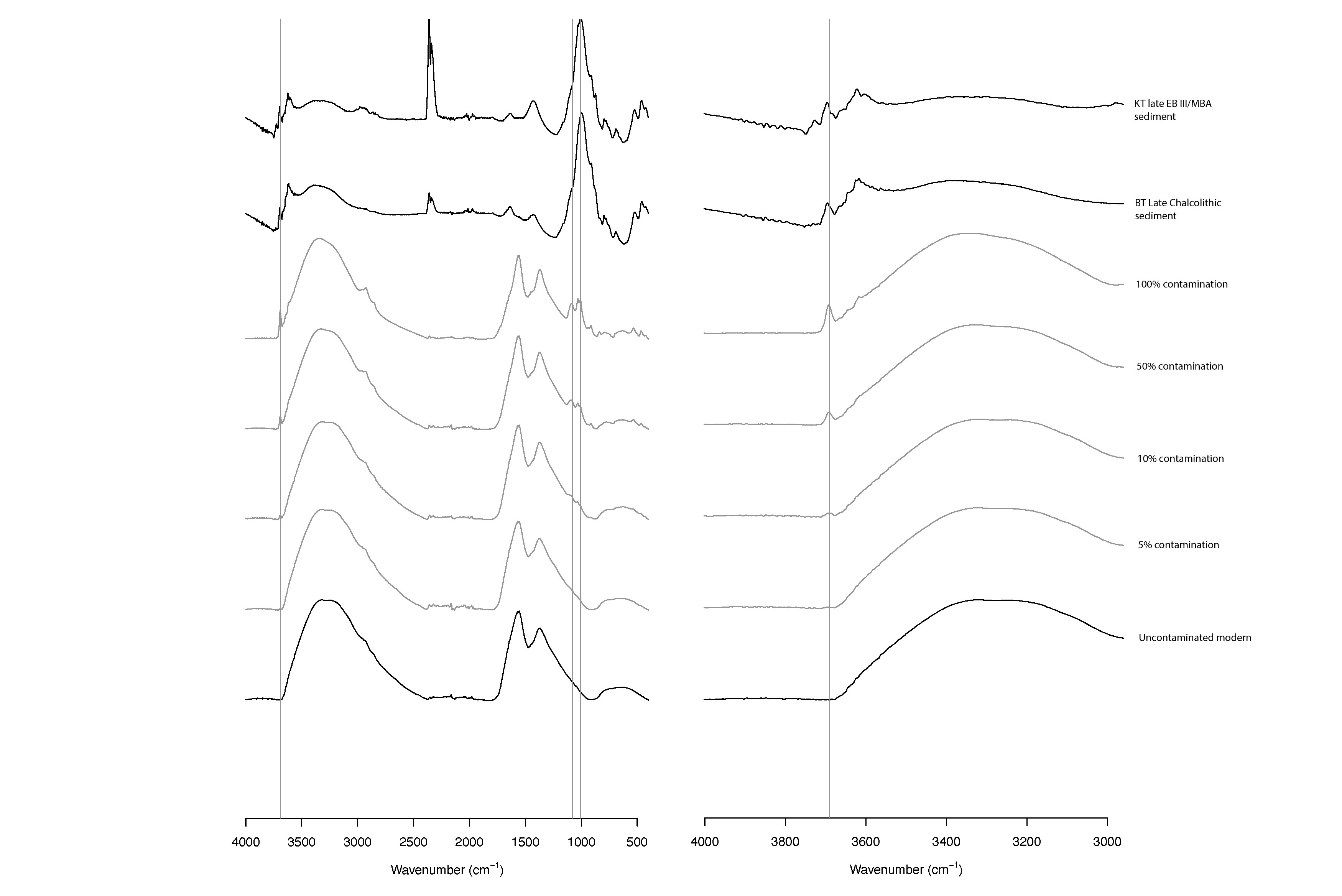


Humics

Humics

**Supplementary Figure 4**. (cont.)

**References**

1. Bronk Ramsey, C. 2009. Bayesian analysis of radiocarbon dates. Radiocarbon, 51 (1). 337-60
2. Bronk Ramsey, C. 2021. OxCal 4.4 Manual. Available: https://c14.arch.ox.ac.uk/oxcalhelp/hlp_contents.html#Introduction Last accessed 4.10.22
3. Reimer, P. *et al*. 2020. The IntCal20 Northern Hemisphere radiocarbon age calibration curve (0–55 cal kBP). Radiocarbon, 62.
4. Şahoğlu, V. 2016. ‘Early Bronze Age Cemeteries at Bakla Tepe: Changing Patterns’. In: Pernicka, E., Ünlüsöy, S. and Blum, S. W. E. (eds), Early Bronze Age Troy: Chronology, Cultural Development and Interregional Contacts. Bonn: Verlag. 167-82
5. van der Veen, M. 2007. ‘Formation processes of desiccated and carbonized plant remains – the identification of routine practice’. Journal of Archaeological Science, 34. 968-90
6. Fraser, R. A. *et al.* 2013. ‘Assessing natural variation and the effects of charring, burial and pre-treatment on the stable carbon and nitrogen isotope values of archaeobotanical cereals and pulses’. Journal of Archaeological Science, 40. 4754-66
7. Szpak, P. and Chiou, K. L. 2019. ‘A comparison of nitrogen isotope compositions of charred and desiccated botanical remains from northern Peru’. Vegetation History and Archaeobotany, 29. 527-38
8. Hartman, G. *et al.* 2020. ‘Post-charring diagenetic alteration of archaeological lentils by bacterial degradation’. Journal of Archaeological Science, 117. 105-19
9. Vaiglova, P., Snoeck, C., Nitsch, E., Bogaard, A. and Lee-Thorp, J. 2014. ‘Impact of contamination and pre-treatment on stable carbon and nitrogen isotopic composition of charred plant remains’. Rapid Communications in Mass Spectrometry, 28. 2497-510
